# The Trail Less Traveled: Individual Decision-Making and Its Effect on Group Behavior

**DOI:** 10.1371/journal.pone.0047976

**Published:** 2012-10-24

**Authors:** Michele C. Lanan, Anna Dornhaus, Emily I. Jones, Andrew Waser, Judith L. Bronstein

**Affiliations:** 1 Department of Entomology, the University of Arizona, Tucson, Arizona, United States of America; 2 Department of Ecology and Evolutionary Biology, the University of Arizona, Tucson, Arizona, United States of America; 3 Department of Entomology, Washington State University, Pullman, Washington, United States of America; 4 Tucson, Arizona, United States of America; Arizona State University, United States of America

## Abstract

Social insect colonies are complex systems in which the interactions of many individuals lead to colony-level collective behaviors such as foraging. However, the emergent properties of collective behaviors may not necessarily be adaptive. Here, we examine symmetry breaking, an emergent pattern exhibited by some social insects that can lead colonies to focus their foraging effort on only one of several available food patches. Symmetry breaking has been reported to occur in several ant species. However, it is not clear whether it arises as an unavoidable epiphenomenon of pheromone recruitment, or whether it is an adaptive behavior that can be controlled through modification of the individual behavior of workers. In this paper, we used a simulation model to test how symmetry breaking is affected by the degree of non-linearity of recruitment, the specific mechanism used by individuals to choose between patches, patch size, and forager number. The model shows that foraging intensity on different trails becomes increasingly asymmetric as the recruitment response of individuals varies from linear to highly non-linear, supporting the predictions of previous work. Surprisingly, we also found that the direction of the relationship between forager number (i.e., colony size) and asymmetry varied depending on the specific details of the decision rule used by individuals. Limiting the size of the resource produced a damping effect on asymmetry, but only at high forager numbers. Variation in the rule used by individual ants to choose trails is a likely mechanism that could cause variation among the foraging behaviors of species, and is a behavior upon which selection could act.

## Introduction

In complex systems such as social insect colonies, the actions of individuals combine to produce emergent behaviors of the group. These collective behaviors are often adaptive [1], [2]. However, they did not necessarily evolve in this context; they may also be epiphenomena that are the consequence of constraints on behavior at the level of the individual [3]. Social insects exhibit a variety of complex colony-level behaviors, including group foraging raids [4], traffic lane formation [5], and construction of nests [6]. Many of these behaviors are of a clear benefit to the colony. However, group foraging, which is not centrally directed but rather emerges from individual foraging decisions, can sometimes result in a colony ignoring other potentially profitable resources when recruitment to one site is strong.

**Figure 1:**
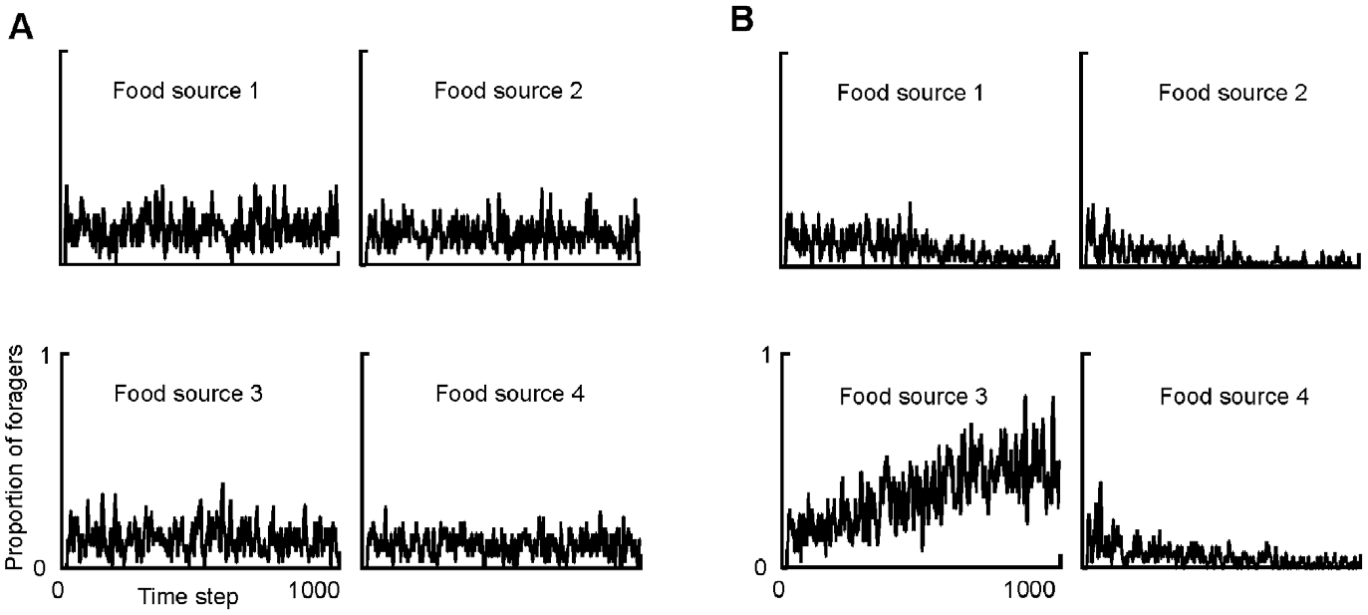
Typical output of two simulations of ants foraging at 4 identical food sources 1–4 (model described in text). In simulation a), symmetry breaking does not occur, and asymmetry (calculated using Equation 2) is 0.065. In simulation b), symmetry breaking occurs, and asymmetry is 0.570. Simulation a) had 500 potential foragers and used the linear choice rule, while simulation b) had 500 potential foragers and used the sigmoidal curve choice rule with *k* = 0 and *x* = 2.

The phenomenon of asymmetrical use of identical resources by a social insect colony has been termed “symmetry breaking” [7], after the more general phenomenon in physics [8]. The mechanism responsible for the shift between symmetrical and asymmetrical use of identical resources is positive feedback in forager recruitment [9], [10]. Stochastic variation in the numbers of foragers that initially encounter each food source results in small differences in recruitment to each source. These differences are amplified as more of the foragers visit the source with stronger recruitment (Fig. 1), sometimes even resulting in complete abandonment of one source in favor of the other.

Symmetry breaking has been demonstrated to occur under laboratory conditions in a number of ant species that use pheromone trail recruitment while foraging [9], [11], [12], [13], [14], [15], [16], [17]. In contrast, no cases of symmetry breaking have been reported in foraging honeybees [18], which use dance recruitment to food patches. The difference in collective foraging behavior between ants and bees has been attributed to the level of non-linearity in the response of individuals to recruitment signals [2], [18]. Given a choice between two pheromone trails that vary slightly in concentration, ant workers exhibit a non-linear response [10], [19], amplifying the difference between the trails. In contrast, honeybee foragers do not compare between dancers recruiting for different food patches, yielding a linear response to the strength of the recruitment signal [20], [21]. This small difference in individual behavior determines whether colonies will forage asymmetrically among identical resources.

Although previous authors have pointed out the importance of non-linear recruitment on symmetry breaking in social insects e.g., [2], [18], the effect of varying the linearity of recruitment response has not been systematically tested. When presented with competing recruitment stimuli, there are a variety of possible mechanisms a forager could use to make a choice, for instance by ranking the options or by responding proportionately to signal strength. In addition to the overall degree of non-linearity, the specific details of this choice mechanism are likely to affect colony-level behaviors such as symmetry breaking, ultimately determining whether colony foraging effort will focus on a few or many sites [18]. The size of the resource patches and the number of foragers (colony size) may also play a role in constraining collective foraging behavior [22]. Individual behavior such as response to recruitment is a key trait on which natural selection can act, shaping the types of collective behaviors exhibited by whole colonies. Forager number is also an important factor influencing collective behavior, one that is correlated with colony size and determined both by natural selection and by colony age and resource constraints. Here, we use stochastic modeling to systematically test how symmetry breaking is affected by the degree of non-linearity of recruitment, the specific mechanism used by individuals to choose a trail, patch size, and forager number, in order to tease apart the effects of these different factors on collective behavior.

## Model

**Figure 2:**
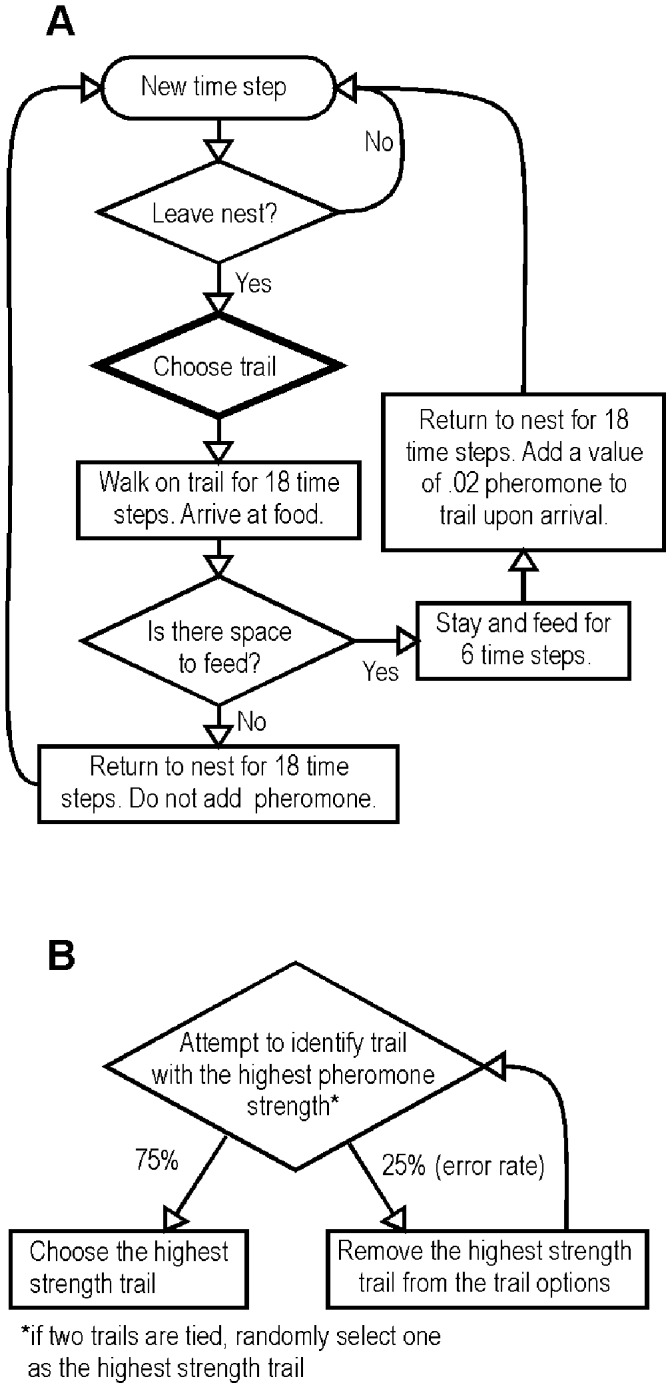
Flow charts describing a) the basic individual-based model, and b) the ranked decision rule. The chart in b) shows the “Choose trail” box from a) in more detail for the ranked rule only.

We implemented and analyzed an individual-based model of ant foraging using the Visual Basic.NET programming language (VB.NET, Microsoft Corp. 2008). In the model, a colony of ants forages at four identical food patches over a period of 1000 time steps. This four-patch design is based on the experimental setup used in our empirical studies of symmetry breaking in ants [23], [24], but the results can easily be related to studies that use a two-patch design (method described below). The basic structure of the model is illustrated in Fig. 2A.

In any given time step, each ant in the nest has a 0.01 probability of leaving the nest to forage. This parameter value causes 25% to 30% of the potential foragers to be outside the nest at any given time in all simulations. Once she leaves the nest, a forager encounters the paths to four food sources and chooses a path using one of three different decision rules that vary in linearity (described below). After walking on the trail and encountering the food source, each ant can either stay and feed or return directly to the nest, based on whether the patch is already filled with ants to its capacity. We tested two food patch sizes: limited patches that accommodated only 15 ants at a time, and unlimited patches. Unlimited patches are meant to represent large food sources, while limited patches are analogous to small food sources with limited access such as extrafloral nectaries [25]. In the model, only ants that successfully feed and return to the nest add pheromone to the trail used, increasing the strength of the trail by 0.02 pheromone units, similar to the pheromone-laying behavior of many ant species [2], [26], [27]. Evaporation of the pheromone over time is captured by multiplying the overall strength of each trail by a decay rate of 0.1 and subtracting this amount from the overall strength at each time step, after all behaviors for that step have been completed. The ants in the model take 18 time steps to reach food patches after choosing a trail, and feed for 6 time steps before returning to the colony. These numbers are based on the timing of behaviors we observed in the ant *Forelius pruinosus* foraging at baits (Lanan, *unpublished data*), where each time step is equivalent to 5 seconds. We performed sensitivity analyses on the time step values and decay rate, which are summarized in the Supporting Information (Fig. S1). These analyses indicated that the behavior of the model was robust to variation in these parameters.

For all sets of parameters tested, we ran 100 simulations for each of 25 colony sizes ranging from 5 to 2000 ants, yielding average active forager numbers ranging from 1 to 600. In addition, for our analyses of the effect of varying discrimination ability (parameter described below) on the behavior of the model at small forager numbers, we ran 500 simulations for 20 colony sizes ranging from 5 to 200.

### Choice Rules

**Figure 3:**
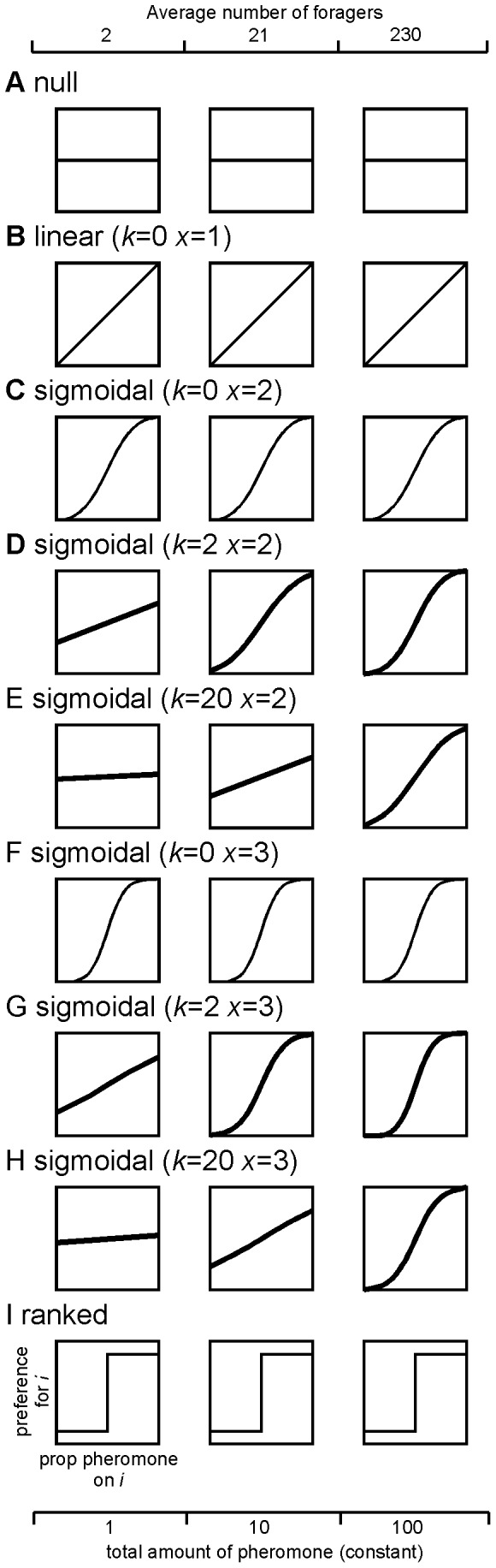
Detailed behavior of choice rules with respect to the relationship between an ant’s preference for a trail and the proportion of pheromone on that trail. A) null model, B) linear choice rule, C) *k* = 0 *x* = 2, D) *k* = 2 *x* = 2, E) *k* = 20 *x* = 2, F) *k* = 0 *x* = 3, G) *k* = 2 *x* = 3, H) *k* = 20 *x* = 3 I) ranked rule.

We ran simulations using choice rules for foragers deciding between trails that varied from completely linear to extremely non-linear responses to recruitment (Table 1). These rules are described below, in order from most to least linear. In all cases we used a random choice rule for comparison (Table 1; Fig. 3A), and in all cases choices in the model were made according to their calculated probability using computer generation of pseudo-random numbers.

**Table 1:** Choice rules used in the simulations.

Rule	Description
**Random choice rule** (null model)	Ants choose trails randomly, never add pheromone, and have a 1/*n* chance of choosing each trail. *n* = 4 for all simulations in this study
**Linear choice rule**	Preference for each trail is directly proportional to the amount of pheromone it contains
**Sigmoidal curve choice rule**	Preference for each trail is calculated using Equation 1
**Ranked choice rule**	Ants rank trails by strength, and choose the strongest with 75% probability (25% error rate). If the strongest trail is not chosen the worker then uses the same decision-making process to choose between the remaining trails. (Fig. 3B)

Using the **linear choice rule**, the preference of an individual ant for a trail was directly related to the proportion of the total pheromone the trail contained (Fig. 3B). If all four trails were identical, an ant would have a 25% chance of taking each trail. Similarly, if one trail contained 75% of the total pheromone, an ant would have a 75% chance of choosing that trail. This choice rule is similar to models of honeybee recruitment [20], [21], in which recruitment response is also linear.

We created varying degrees of non-linearity in ant response to recruitment using the **sigmoidal choice rule**. A sigmoidal curve has been used previously e.g., [10], [22] to describe the preference of an individual Argentine ant (*Linepithema humile*) worker for a trail based on the amount of pheromone on it. If *c_i_* is the amount of pheromone on trail *i*, then the preference for trail *i* (i.e., the probability the ant will take trail *i*) is described as
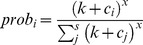
(1)where *s* is the total number of trails. This formula produces a sigmoidal curve, the specific shape of which is determined by the constants *x* and *k.* The parameter *k* indicates the attractiveness of trails to workers regardless of the strength of the pheromone; thus, the higher the *k* value, the greater the level of trail laying necessary for symmetry breaking to occur [2]. The parameter *x* affects the preference of the ants for trails with greater amounts of pheromone, such that even when the difference among trails is small, ants will have a high likelihood of choosing the stronger trail when *x* is large [2]. In terms of the preference curve, increasing *k* causes a decrease in the upper asymptote of the curve and an increase in the y-intercept, while increasing *x* causes an increase in the steepness or non-linearity of the curve (Fig. 3 C–H). Versions of this sigmoidal choice curve have been used previously in a number of differential equation [10], [22], [28], [29], [30] and simulation [4], [16], [19], [31], [32] models of collective behavior.

It is important to note that Eq.1 generates a preference curve using the total *amount* (*c_i_*) rather than *proportion* of pheromone on the trails, such that the effect of pheromone on a trail depends on its strength relative to the intrinsic attractiveness of the trails (*k*). Thus, if we plot the preference for trail *i* against the proportion of pheromone on trail *i*, we see that in some cases the shape of the preference curve changes depending on the total quantity of pheromone present. Because the total amount of pheromone that accumulates on the trails is dependent on the number of foragers, the shape of the preference curve can therefore change as colony size increases, as well as changing as pheromone accumulates over time (Fig. 3 D, E, G, H). We discuss the effect of this changing curve shape on symmetry breaking in the results.

In our study we use six sets of parameters for the sigmoidal curve that vary the degree of linearity in trail choice (Fig. 3 C–H). We use three values of *k* ranging from low to high (*k = *0, 2, 20) and two values of *x* (2 and 3). When *k = *0 (i.e., trails have no intrinsic attractiveness), the shape of the preference curve remains constant across all forager numbers (Fig. 3 C, F). However, when *k >*0 the curve becomes increasingly non-linear as forager number and pheromone amount increases. High values of *k* produce the most linear curves with the highest y-intercepts at low forager numbers, although they become similar to the curves produced by *k = *0 at high forager numbers as the intrinsic attractiveness of the trails become insignificant relative to the strong pheromone signal (e.g. Fig. 3 E, H). Increasing the parameter *x* increases the non-linearity of the curve as well. It is worth noting here that the linear choice rule described above (Fig. 3 B) is a special case of the sigmoidal rule with *k = *0 and *x = *1. Previous experimental studies have determined the value of *x* and *k* for two species of ants, *Linepithema humile* (*k = *20 and *x = *2), [10] and *Lasius niger* (*k = *6 and *x = *2), [19]. In empirical studies such as these, the observed preference curve, and thus the values of *x* and *k* estimated from it, may reflect not only individual preferences, but also the experimental conditions such as substrate type in which ants are tested. However, in our model the environment does not vary and so the curve describes only individual choice behavior, which is sometimes dependent on forager number.

The third rule we tested, the **rank choice rule**, produces the most extreme non-linearity of ant response to trail pheromone (Fig. 3 I). Using this rule, individual ants rank the trails by strength and then choose the strongest trail with a 25% error rate (i.e., ants randomly choose among one of the three weaker trails 25% of the time). Ties between trails with similar pheromone values were broken using the procedure shown in Fig. 2 B. A similar rank choice rule has been used previously in two Monte-Carlo type simulation studies that describe ants making a trail through 2D space [15], [33], although this choice model has not previously been compared to other models of symmetry breaking in social insects. Ant workers might use such a rule if they are most attracted to the strongest trail they detect, regardless of the strength of competing trails.

None of the simulations described here included individual memory for the location of food sources. Memory plays an important role in foraging in many ant species (e.g., [25], [34], [35], [36]. However, due to the wide variety of ways in which memory could affect the system, we ignore it here in order to focus on the effects of varying the linearity of individual choice rules.

### Discrimination between Trails

The basic version of the model assumes that ants can discriminate among trails that vary in infinitesimally small amounts of pheromone. However, in nature it is likely that ants can not discriminate among trails that are very similar in pheromone concentration [37]. To explore the effect of discrimination ability on the behavior of the model, we added a discrimination value that varied from 0 to 5, representing the minimum difference between quantities of pheromone that an ant can detect. A high value therefore represents coarse discrimination ability, while a low value represents fine discrimination ability. Hereafter in the text, simulations with a discrimination value of 5 are referred to as having ‘coarse’ discrimination ability, while simulations using a value of 0 are referred to as having ‘fine’ discrimination ability. Discrimination ability in our model is not equivalent to the effect of *k* in the sigmoidal choice rule, which rather describes the baseline attractiveness of all trails.

In our model, the discrimination value was applied before the decision rule, so that trails that varied by an amount below the discrimination value were treated as equal. In simulations using the ranked choice rule, trails that differed by an amount below the discrimination value were ranked equally, and when using the linear and sigmoidal choice rules, such trails were treated as though they had equal (averaged) amounts of pheromone.

### Analysis

To describe how asymmetrically each group of ants foraged over time in each simulation, we calculated the average proportion of ants that visited the most preferred food source, *a*. We then standardized this value so that it ranged from 0 to 1, using the formula
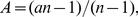
(2)in which *n* is the number of food sources a colony could visit. In our simulations *n* = 4. The value *A* is a dimensionless index of asymmetry used to compare between simulations and is not a measure of the proportion of foragers on a trail. Because asymmetry (*A*) calculated using this formula is independent of the number of food sources, it can be used in future work to standardize comparisons of foraging asymmetry between experiments with varying numbers of food sources.

In order to capture the behavior of the model once ant recruitment had stabilized (typically within the first 50–70 time steps), only the last 900 of the 1000 time steps simulated were used to calculate asymmetry. Because the asymmetry value is bounded by 0 and 1 and the distribution of the model output was frequently non-normal, we used non-parametric statistics for comparing output of the different models and the null hypothesis. However, in order to describe the direction of the relationship between asymmetry and forager number we used linear regression for all models.

## Results

### Effect of Non-linearity of the Choice Rule

**Figure 4:**
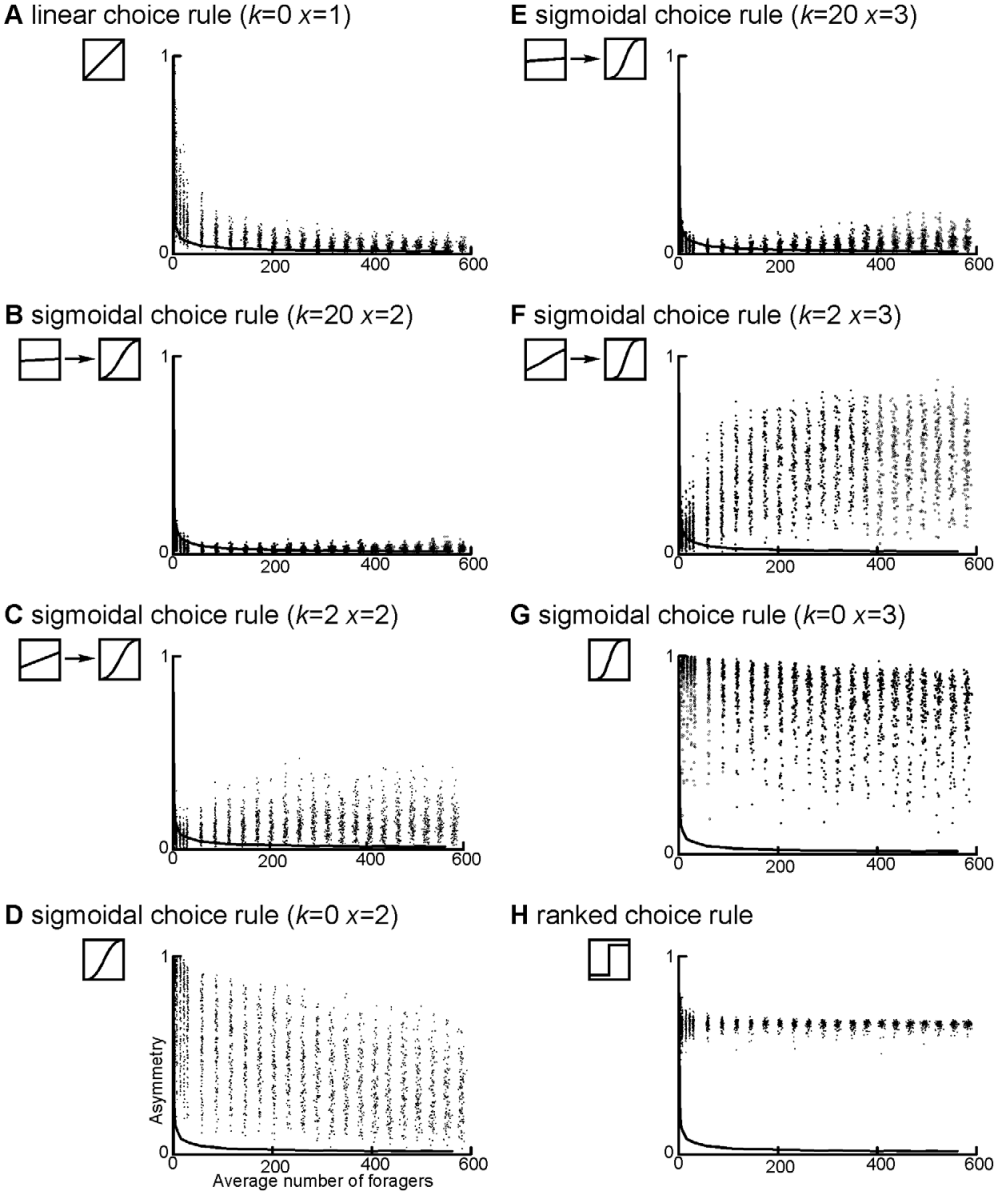
Asymmetry increases as the linearity of the decision rules decreases. In this figure we show the relationship between average forager number and asymmetry for a) the linear choice rule, b) the sigmoidal choice rule with *k = *20 and *x = *2, c) with *k = *2 and *x = *2, and d) with *k = *0 and *x = *2, e) with *k = 2*0 and *x = *3, f) with *k = 2* and *x = *3 g) with *k = *0 and *x = *3 and h) the ranked choice rule. The upper 95% quantile of the null is shown as a solid line on all graphs; thus all points above show significantly higher asymmetry than a model without pheromone trails. The insets show the relationship between the probability that an ant will choose a trail (*prob_i_*) and the proportion of total pheromone on that trail (*c_i_*), for the choice models a–h. Choice models in which the preference changes with pheromone quantity are represented by two graphs (low and high pheromone) connected by an arrow.

Foraging symmetry was strongly affected by the trail choice rule used, although in every case the rules produced asymmetry significantly greater than the null model (equal preference for all trails) at all forager numbers (Fig. 4, Table 2). The least asymmetry was produced by the sigmoidal choice rule with *k* = 20 and *x* = 2 (*Ā = *0.026, Fig. 4 B), followed by the linear rule (*Ā = *0.034, Fig. 4 A) and the sigmoidal rule with *k* = 20 and *x* = 3 (*Ā = *0.045, Fig. 4 F). These two sigmoidal curves with high *k* values were nearly linear with high y-intercepts at low forager numbers, although they resembled the other non-linear sigmoidal curves at high forager numbers (Fig. 3 F, J). In contrast, the consistently non-linear rules (e.g., the ranked choice rule and the sigmoidal choice rule with *k* = 0 and *x* = 2 or 3) produced mean asymmetry values that were much higher (*Ā* >0.5) than the null model (Fig. 4 E, I, J). To further test the effect of increasing non-linearity, we varied just the exponent *x* from 2 to 5 with *k* constant at 0. As expected, mean asymmetry in these simulations increased with increasing *x* (Linear Regression, F_1,9999_ = 7992.074, *P*<0.0001).

**Table 2:** Effects of the different choice rules on the simulations.

Choice rule	Median asymmetry	Relationship between asymmetry and forager number	Difference between asymmetry and null for highest observed asymmetry value	Spread of asymmetry (average difference between upper and lower 95% quantiles)
Linear *k* = 0, *x* = 1	0.034	Decreasing*	0.553*	0.174
Sigmoidal *k* = 0, *x* = 2	0.604	Decreasing*	0.917*	0.427
Sigmoidal *k* = 2, *x* = 2	0.336	Increasing*	0.154*	0.212
Sigmoidal *k* = 20, *x* = 2	0.026	Increasing*	0.060*	0.054
Sigmoidal *k* = 0, *x* = 3	0.844	Decreasing*	0.882*	0.482
Sigmoidal *k* = 2, *x* = 3	0.362	Increasing*	0.802*	0.456
Sigmoidal *k* = 20, *x* = 3	0.045	Increasing*	0.157*	0.085
Ranked	0.662	No relationship	0.563*	0.078

The different choice rules produced differing median asymmetry values, and also differed in the relationship between forager number and asymmetry and the spread of asymmetry values produced. We determined the greatest difference between asymmetry and the null by comparing the highest observed asymmetry value with the corresponding value in the null model. All relationships between asymmetry and forager number marked with a star are significant (Linear Regressions, all *P*<0.0001), and all differences between the highest asymmetry and the null were significant (Wilcoxon Rank-Sum tests, all *P*<0.0001).

The rules also differed greatly in how much variation in asymmetry occurred between simulations (Table 2), with the greatest variation occurring in the least linear sigmoidal simulations (Fig. 4 D, E). The wide range of values produced by the model suggests that stochastic variation can lead to a whole range of colony-level behaviors, even when individual recruitment response is highly non-linear. For example, a small percentage of simulations with *k* = 0 and *x* = 3 produced results that were only weakly asymmetrical, with *A* values around 0.1.

### The Parameter k and Forager Number

**Figure 5:**
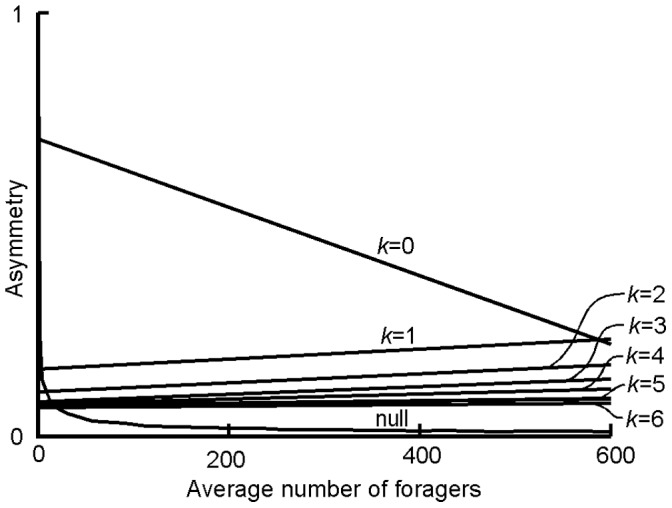
The linear regression lines for simulations using *k* = 0, 1, 2, 3, 4, 5, and 6, when *x* = 2, patch capacity is unlimited, and discrimination ability is infinite. The upper 95% quantile of the null is included, and for all linear regressions shown, *P*<0.0001.

The parameter *k* represents the baseline attractiveness of all trails in the sigmoidal choice rule, and increasing *k* increases the y-intercept and reduces the upper asymptote of the curve at low forager numbers. This high y-intercept increases the probability that trails with low pheromone will be chosen, reducing asymmetry at low forager numbers. To better demonstrate the effect of increasing *k* on the behavior of the model at different colony sizes, we plotted linear regression lines from sets of simulations using *k* = 0, 1, 2, 3, 4, 5, and 6 with *x* = 2 (Fig. 5). Using *k* = 0 produced a special case in which asymmetry decreased with increasing forager numbers, due to the non-linear shape of the preference curve even at low amounts of pheromone (Fig. 3C). When *k = *0, preference depends only on the proportion of pheromone present on a given trail and thus the shape of the preference curve remains constant. For all other values of *k,* preference is dependent on the amount of pheromone and the relationship between asymmetry and forager number has a positive slope. As *k* increases from 1 to 6, the slope of this relationship decreased (Linear Regression, F_1,5_ = 363.556, *P*<0.0001) due to the increasing intrinsic attractiveness of the trails.

In simulations using the ranked rule, we observed no relationship between forager number and asymmetry, and the mean asymmetry across all forager numbers was approximately 0.66, which is equal to the transformed (Eq. 2) value of the set preference for the highest ranked trail (0.75). Sensitivity analyses for this rule indicated that average asymmetry was always approximately equal to transformed preference, regardless of the value used (Supporting Information, Fig. S2). Interestingly, at very low forager numbers the rank choice rule produced lower asymmetry values than the sigmoidal rule with *k = *0 and *x = *2 or 3. This is due to the higher y-intercept of the rank choice model relative to the sigmoidal rule with *k = *0.

### Effect of Discrimination Ability

**Figure 6:**
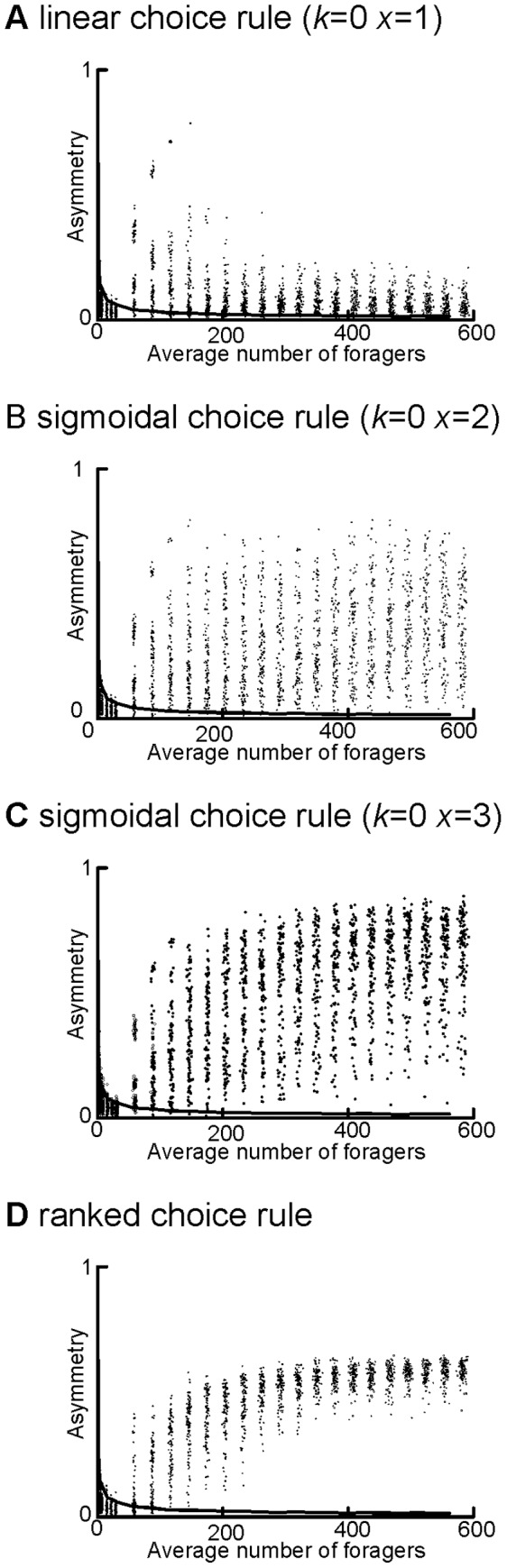
Discrimination ability and asymmetry. Limiting the ability of individual ants to distinguish between trails with similar amounts of pheromone (discrimination = 5) caused asymmetry to become indistinguishable from the null at low average forager numbers for a) the linear choice rule, b) the sigmoidal choice rule with *k = *0 and *x = *2, c) the sigmoidal choice rule with *k = *0 and *x = *3, and d) the ranked choice rule. The upper 95% quantile of the null is shown as a solid line on all graphs.

Limiting the ability of ants to discriminate between trails with similar amounts of pheromone created a threshold number of foragers below which the colonies’ degree of foraging asymmetry did not significantly differ from the null model (with no recruitment), but above which colonies foraged asymmetrically (Fig. 6, Wilcoxon Rank-Sum Tests, *P*<0.05 for all choice rules). This threshold forager number increased linearly with the size of the minimum difference in pheromone that workers could detect (Linear Regression, F_1,5_ = 1011.434, *P*<0.0001).

### Effect of Patch Capacity

**Figure 7:**
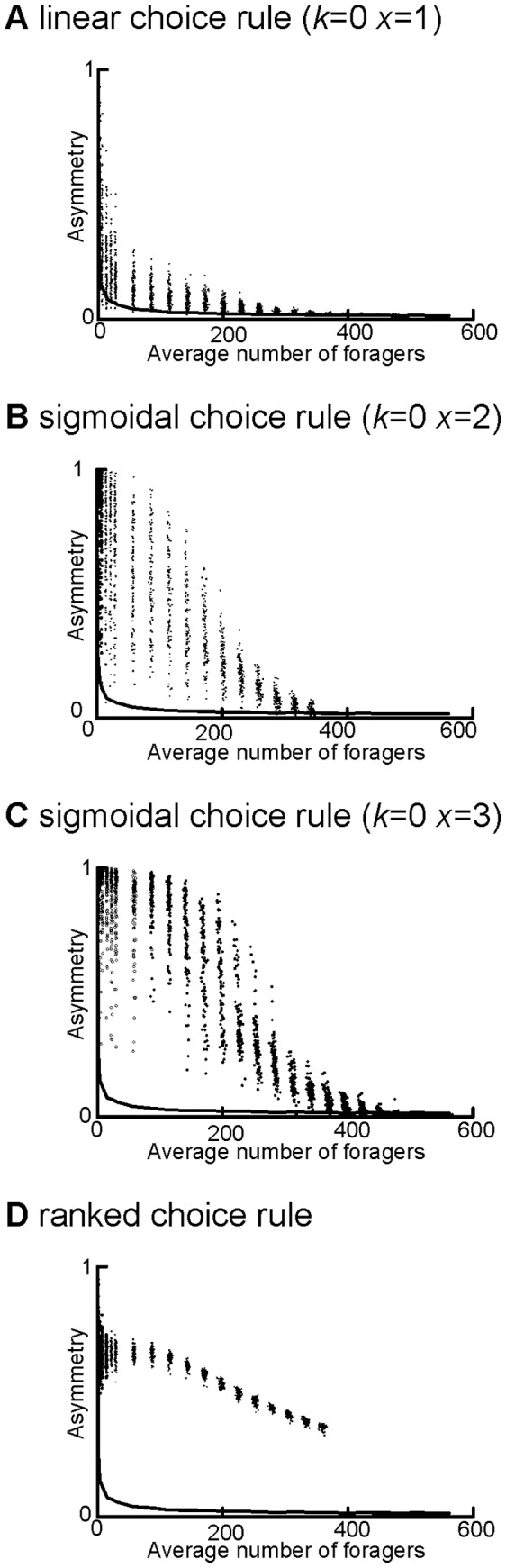
Asymmetry decreased at large average forager numbers when patch size was limited for a) the linear choice rule, b) the sigmoidal choice rule with *k = *0 and *x = *2, c) the sigmoidal choice rule with *k = *0 and *x = *3, and d) the ranked choice rule. The upper 95% quantile of the null is shown as a solid line on all graphs.

Regardless of the choice model used, limiting the capacity for ants at the patches produced a damping effect on asymmetry as average forager number increased (Fig. 7). With all choice models, asymmetry declined above a threshold number of ants, and all simulations except those produced using the ranked choice rule became indistinguishable from the null model at high forager numbers (for detailed statistical analyses, see Supporting Information, Table S1).

## Discussion

Symmetry breaking in ants can cause strongly asymmetrical patterns of foraging among food patches, a condition that could lead a colony to incur either costs or benefits depending upon ecological conditions. Previous authors [2], [18] have predicted that asymmetry in foraging is determined by the non-linearity of individual choice behavior in response to the strength of signals for different patches. These previous studies have mainly focused on comparing the moderately non-linear recruitment of the ants *Lasius niger* and *Linepithema humile* to the linear behavior of honeybees [2], [18]. Here, we have compared all the proposed choice models using stochastic simulations of ant foraging. We show that the degree of non-linearity is a key element in determining the level of asymmetry exhibited by foraging social insects, while the amount of variability in foraging asymmetry is influenced by details of the decision-making process. Our results also demonstrate that foraging asymmetry can be influenced by pheromone quantity, a factor dependent on forager number (i.e., colony size).

It is clear that symmetry breaking can either be enhanced or mostly avoided through the modification of individual response to recruitment stimuli. Selection on individual behaviors such as the choice rules we examined here is therefore likely to be one mechanism accounting for variation among the collective foraging behaviors of different ant species. Variation within species, such as differences between foraging behavior of young and old colonies, could also be due to changes in individual choice behavior. Nevertheless, our simulations revealed a surprising degree of variation in foraging asymmetry produced by replicate runs of individual decision rules. Although simulations using the least linear sigmoidal rules produced highly asymmetrical foraging on average, individual simulations occasionally produced only moderate or low levels of symmetry breaking. Thus, we would expect that in the field, even ants with extremely non-linear responses to recruitment stimuli may occasionally forage symmetrically if they use a sigmoidal choice rule. In contrast, the very non-linear rank choice rule produced only small amounts of variation, highlighting the role of behavioral details in determining variability. These results demonstrate the advantage of simulations over differential equation models in predicting certain aspects of collective behaviors in complex systems, as differential equation models do not capture the full variation of stochastic processes.

We also used our simulations to investigate whether the degree of asymmetry produced by these different choice models is sensitive to additional characteristics of a species and its resources. Importantly, we found that the choice models differ qualitatively in how foraging asymmetry is altered by these factors. In particular, forager number changes the effects of the relative non-linearity of the choice model. At low forager numbers, and thus low total pheromone concentrations, it is the intrinsic attractiveness of trails (or error rate, in the case of the rank choice model), rather than the non-linearity of the choice rule, that has the greatest role in determining foraging asymmetry. Models with intrinsically attractive trails produce low foraging asymmetry, whereas models lacking intrinsic attraction to trails produce high foraging asymmetry. As forager number increases, foraging asymmetry can increase, remain the same, or decrease. We have shown that in the sigmoidal choice model [10], [22] the non-linearity of individual choices actually increases with forager number when trails are intrinsically attractive (i.e., *k*>0). Since high forager numbers increase the strength of the pheromone signal relative to the intrinsic attractiveness of the trails, foraging asymmetry also increases with forager number. The absolute strength of the pheromone signal does not affect the other choice rules, and in the rank-choice model there is no effect of increasing forager number. In the remaining choice models, asymmetry decreases with increasing forager number, likely due to the lower initial asymmetry between trails and slower loss of weaker trails when forager numbers are high.

A second effect of low forager numbers occurred when individuals had only a coarse ability to distinguish between trails with similar pheromone concentrations. In this case, there was a threshold number of ants below which all choice models produced symmetric foraging patterns. This threshold effect is similar to the relationships between forager number and asymmetry previously reported for several ant species. *Tetramorium casepitum* only exhibits symmetry breaking over a threshold colony size, below which they forage symmetrically [11]. Similarly, the foraging behavior of *Monomorium pharaonis* undergoes a ‘phase transition’ from individual foraging to mass trail recruitment as colony size increases [38]. This transition is also predicted by a model of ant behavior [22]. Interestingly, in these studies, the abrupt change in colony-level foraging behavior occurred simply due to changing group size, despite the unchanged behavior of individual ants [38]. In our study a similar threshold occurred when we limited the discrimination ability of the ants, and a threshold-like effect was also produced using the sigmoidal rule with *k* >0. It would be difficult to discriminate between patterns produced by these different rules using empirical studies of ant foraging such as those described by [9], [38].

At high forager numbers, the size of patches became an important factor influencing the asymmetry produced by the different choice models. In all choice models, above a threshold number of foragers, foraging was more symmetric when patch size was limited. Since only those individuals that successfully foraged at a patch added to the pheromone trail, positive feedback in trail strength was limited once a patch reached capacity and ants that did not find space to forage turned back. In nature, small resource patches with limited access are common, such as extrafloral nectaries situated on slender stems with limited flow rate. Colonies may also alter collective foraging behavior in response to patch characteristics, for instance by increasing the time individuals spend feeding [39] or by changing the rate of recruitment from the nest [40].

In this study we incorporated a single method of introducing non-linearity to a model of ant colony behavior by modifying the choice rule used by individuals. However, many other non-linear mechanisms have also been demonstrated to influence the collective behavior of social insects. Workers can change their behavior in ways that influence colony foraging, for instance by modulating trail-laying behaviors such as the amount of pheromone used [41], [42]. Crowd following, in which the decision of one individual is mimicked and thus amplified by those around it, is another behavior that has been demonstrated to lead to symmetry breaking in both ants and cockroaches [43], [44]. U-turns made by ants walking on trails can play an important role in determining how quickly individual trails are enhanced, affecting the emergence of asymmetry [45]. Behavioral mechanisms also exist that can prevent asymmetry when it is maladaptive for the colony. For instance, collisions between ants walking on a narrow Y-shaped bridge lead to a more symmetrical and thus more efficient flow of traffic across the bridge [46].

What is the adaptive significance of symmetry breaking in foraging ants? In a natural environment, ant colonies are unlikely to encounter resources that are truly identical in quality, distance, and retrievability. However, the same mechanisms that result in symmetry breaking in the laboratory enable colonies to make collective decisions among resources that differ. For instance, recruitment to superior food sources will usually occur at a faster rate, enabling colonies to choose the higher quality source [17], [31]. A similar feedback mechanism also enables colonies to choose the shortest route to a food source [16]. Workers are also less likely to become lost while walking on a single strong trail that arises through symmetry breaking, rather than on several weak trails [12], [22]. In addition, the ability to recruit numerous workers to one food source quickly may enable ants to repel competitors, as competitive interactions between colonies are often resolved through numerical dominance [26], [47].

Nevertheless, the relative advantages of symmetry breaking are likely to depend on the requirements of each species e.g., [24]. Strong symmetry breaking may be a mechanism that enables dominant ants to competitively exclude other species from resources, as described above. However, for species that are lower in the dominance hierarchy and that rely on rapid discovery of new food sources, symmetry breaking may be maladaptive, since the tendency to create one strong trail would prevent workers of these species from exploring new areas. Furthermore, symmetry breaking could also increase predation risk, because larger worker numbers attract more predators such as phorid flies [48], [49]. Symmetry breaking may also exact a cost in the form of reduced information available to colonies. When too great an asymmetry arises, colonies abandon trails to alternate food sources. In species that do not use area marking [50], colonies with lost trails would lose collective knowledge of the food sources and the opportunity to exploit them later. Honey bees are able to avoid this problem by using a linear response to recruitment signals, thus sustaining low-level recruitment to sites that are currently less profitable [13]. We suggest that a more linear recruitment response may also occur in ant species that need to avoid strongly asymmetrical foraging.

## Supporting Information

Figure S1 **The asymmetry values produced across a range of decay values.** The figure shows a) simulations using the linear choice rule and a discrimination value of 5, and b) simulations using the sigmoid curve rule with *k = *0 and *x* = 2 and a discrimination value of 5. Boxes indicate quantiles.
(TIF)

Figure S2 **The mean asymmetry produced by the ranked rule was always equal to the transformed (Eq. 2) value of the set preference for the highest ranked trail.** For example, when preference was set to 0.75 the mean asymmetry was approximately 0.66, equal to 0.75 transformed by Eq. 2.
(TIF)

Table S1 **Patch capacity.** The average number of foragers at which simulations with patch capacity 15 diverged from simulations withnlimited capacity (i.e., the point at which asymmetry values were significantly lower), and the number of foragers at which simulations with patch capacity 15 converged with the null (i.e. no significant difference detected).
(DOC)
