# Supplementary material for: The Trail Less Traveled: Individual Decision-Making and Its Effect on Group Behavior
Source: PLoS One. 2012 Oct 24;7(10):e47976. doi: 10.1371/journal.pone.0047976 (PMC3480453; doi:10.1371/journal.pone.0047976)
Supplement: Table S1 — Patch capacity. The average number of foragers at which simulations with patch capacity 15 diverged from simulations withnlimited capacity (i.e., the point at which asymmetry values were significantly lower), and the number of foragers at which simulations with patch capacity 15 converged with the null (i.e. no significant difference detected). (DOC) [file pone.0047976.s003.doc]

**Table S1: Patch capacity.** The average number of foragers at which simulations with patch capacity 15 diverged from simulations withnlimited capacity (i.e., the point at which asymmetry values were significantly lower), and the number of foragers at which simulations with patch capacity 15 converged with the null (i.e. no significant difference detected).

| **Choice rule** | **Divergence point with simulations of unlimited patch capacity** | **Convergence point with null simulations** |
| --- | --- | --- |
| Linear choice rule | 203 foragers  (Wilcoxon Rank-Sum, χ^2^_1_= 10.2924, *P*=0.0013) | 398 foragers  (Wilcoxon Rank-Sum, χ^2^_1_= 3.467, *P*=0.0626). |
| Ranked choice rule | 90 foragers  (Wilcoxon Rank-Sum, χ^2^_1_= 4.8466, *P*=0.0277). | Always significantly greater than null |
| Sigmoidal curve choice rule, *k*=0 | 146 foragers  (Wilcoxon Rank-Sum, χ^2^_1_= 10.324, *P*=0.0013). | 449 foragers  (Wilcoxon Rank-Sum, χ^2^_1_= 0.752, *P*=0.3857). |
| Sigmoidal curve choice rule, *k*=2 | 174 foragers  (Wilcoxon Rank-Sum, χ^2^_1_= 6.482, *P*=0.011). | 427 foragers  (Wilcoxon Rank-Sum, χ^2^_1_=3.367, *P*=0.0665). |
| Sigmoidal curve choice rule, *k*=6 | 174 foragers  (Wilcoxon Rank-Sum, χ^2^_1_= 4.143, *P*=0.0418). | 398 foragers  (Wilcoxon Rank-Sum, χ^2^_1_= 0.646, *P*=0.421). |
